# Relative sensitivity of mice to inhaled ozone and cigarette smoke exposure

**DOI:** 10.1186/1476-9255-10-S1-P27

**Published:** 2013-08-14

**Authors:** Rachel A Armstrong, Stephen M Jordan, Emma McConnell, Robert Carrington, Debbie Rodgers, Kenneth G Meecham

**Affiliations:** 1 Department of Pharmacology, Huntingdon Life Sciences, Cambridgeshire, UK

## 

Inhaled exposure of both the environmental pollutant ozone and cigarette smoke are important risk factors in the development of chronic obstructive pulmonary disease. We assessed the relative pulmonary sensitivity of mice following ozone challenge in comparison to acute cigarette smoke exposure.

For ozone, mice were exposed to a single 3 hour whole body challenge of 3, 7 and 10 ppm. Animals were sacrificed at either 4 or 24 hours post-exposure. For the acute cigarette smoke model, mice (BALB/c and C57BL6) were exposed to 250, 600 and 800 μg/L particulate concentration for 60 minutes twice daily over a four day period. Air was used as control. Animals were sacrificed 24 hours following the final exposure. Recruitment of inflammatory cells to the lung was assessed in BALF by flow cytometry. Inflammatory responses and anti-inflammatory effects of a corticosteroid and PDE4 inhibitor were assessed.

Ozone exposure induced a concentration dependent neutrophilia with a 3-fold increase at 3 ppm (0.19±0.03 v 0.54±0.12 x 10^5^ cells/animal) peaking at 0.73±0.10 x10^5^ cells/animal 4 hours following 10 ppm exposure. Similar neutrophil counts were recorded at 4 and 24 hours post-exposure. Increased BALF macrophage counts were also recorded but only following 10 ppm challenge (0.74±0.11 v 2.44±0.34 x 10^5^cells/animal) at 24 hours post challenge. Intervention with budesonide (at a dose regime that produces 90% inhibition of LPS induced BALF neutrophilia) did not inhibit cellular recruitment.

Repeated exposure to cigarette smoke induced a concentration and time dependent neutrophilia in BALB/c mice reaching statistical significance as early as Day 2 following exposure at 600μg/L. Comparatively C57BL6 mice were insensitive to cigarette exposure with at least a 5-fold lower response at the identical exposure concentrations. Cigarette smoke induced neutrophilia was also steroid-insensitive.

